# Impact of reproductive factors on breast cancer incidence: Pooled analysis of nine cohort studies in Japan

**DOI:** 10.1002/cam4.3752

**Published:** 2021-03-01

**Authors:** Taro Takeuchi, Yuri Kitamura, Tomotaka Sobue, Mai Utada, Kotaro Ozasa, Yumi Sugawara, Ichiro Tsuji, Miyuki Hori, Norie Sawada, Shoichiro Tsugane, Yuriko N. Koyanagi, Hidemi Ito, Chaochen Wang, Akiko Tamakoshi, Keiko Wada, Chisato Nagata, Taichi Shimazu, Tetsuya Mizoue, Keitaro Matsuo, Mariko Naito, Keitaro Tanaka, Manami Inoue, for the Research Group for the Development, Evaluation of Cancer Prevention Strategies in Japan

**Affiliations:** ^1^ Department of Social Medicine Osaka University Graduate School of Medicine Osaka Japan; ^2^ Department of Epidemiology Radiation Effects Research Foundation Hiroshima Japan; ^3^ Division of Epidemiology Department of Health Informatics and Public Health School of Public Health Tohoku University Graduate School of Medicine Sendai Japan; ^4^ Epidemiology and Prevention Group Center for Public Health Sciences National Cancer Center Tokyo Japan; ^5^ Division of Cancer Information and Control Department of Preventive Medicine Aichi Cancer Research Institute Nagoya Japan; ^6^ Department of Public Health Aichi Medical University School of Medicine Nagakute Japan; ^7^ Department of Public Health Faculty of Medicine Hokkaido University Hokkaido Japan; ^8^ Department of Epidemiology and Preventive Medicine Gifu University Graduate School of Medicine Gifu Japan; ^9^ Department of Epidemiology and Prevention Center for Clinical Sciences National Center for Global Health and Medicine Tokyo Japan; ^10^ Division of Cancer Epidemiology and Prevention Aichi Cancer Center Research Institute Nagoya Japan; ^11^ Division of Cancer Epidemiology Nagoya University Graduate School of Medicine Nagoya Japan; ^12^ Department of Oral Epidemiology Graduate School of Biomedical and Health Sciences Hiroshima University Hiroshima Japan; ^13^ Department of Preventive Medicine Faculty of Medicine Saga University Saga Japan; ^14^ Department of Cancer Epidemiology Graduate School of Medicine The University of Tokyo Tokyo Japan

**Keywords:** breast cancer, cancer risk factors, epidemiology and prevention, meta‐analysis

## Abstract

Prior studies reported the association of reproductive factors with breast cancer (BC), but the evidence is inconsistent. We conducted a pooled analysis of nine cohort studies in Japan to evaluate the impact of six reproductive factors (age at menarche/age at first birth/number of births/age at menopause/use of female hormones/breastfeeding) on BC incidence. We conducted analyses according to menopausal status at the baseline or at the diagnosis. Hazard ratio (HR) and 95% confidence interval (CI) were estimated by applying Cox proportional‐hazards model in each study. These hazard ratios were integrated using a random‐effects model. Among 187,999 women (premenopausal: 61,113, postmenopausal: 126,886), we observed 873 premenopausal and 1,456 postmenopausal cases. Among premenopausal women, use of female hormones significantly increased BC incidence (HR: 1.53 [1.04–2.25]). Although P value for trend was not significant for age at first birth and number of births (P for trend: 0.15 and 0.30, respectively), women giving first birth at ages ≥36 experienced significantly higher BC incidence than at ages 21–25 years, and women who had ≥2 births experienced significantly lower BC incidence than nulliparous women. Among postmenopausal women, more births significantly decreased BC incidence (P for trend: 0.03). Although P value for trend was not significant for age at first birth and age at menopause (P for trend: 0.30 and 0.37, respectively), women giving first birth at ages 26–35 years experienced significantly higher BC incidence than at ages 21–25 years, and women with age at menopause: ≥50 years experienced significantly higher BC incidence than age at menopause: ≤44 years. BC incidence was similar according to age at menarche or breastfeeding history among both premenopausal and postmenopausal women. In conclusion, among Japanese women, use of female hormones increased BC incidence in premenopausal women, and more births decreased BC incidence in postmenopausal women.

## 1 INTRODUCTION

Among women, breast cancer is one of the most prevalent cancer types.^1^, ^2^, ^3^ Although its incidence is lower in Asian countries than in Western countries,^4^, ^5^ it has been increasing in Asian countries.^4^ Reproductive factors are one of the possible risk factors.^3^, ^6^, ^7^, ^8^

Although previous epidemiological studies have reported the impact of reproductive factors on breast cancer,^9^, ^10^, ^11^, ^12^, ^13^, ^14^, ^15^ results were inconsistent between studies, especially in Asian countries. Particularly, the impact of age at menarche/breastfeeding history on breast cancer were inconsistent between studies,^9^, ^10^, ^11^, ^12^, ^13^, ^14^, ^15^ mainly because some studies could not conduct stratified analyses according to the menopausal status. The impact of age at menarche on the incidence of breast cancer was evaluated in previous studies,^9^, ^10^, ^11^, ^12^, ^14^, ^15^ but results were inconsistent. Nagata C and colleagues ^9^ and Liu R and colleagues ^15^ reported that breast cancer occurrence was significantly lower among women with age at menarche ≥16 years than those with ≤13 years. Iwasaki M and colleagues ^11^ reported that breast cancer incidence was significantly lower among women with ≥16 years than those with <14 years. However, other studies in Asia ^10^, ^12^, ^14^ reported that the incidence did not change according to age at menarche. The association between the history of breastfeeding and breast cancer was also inconclusive. Pooled analysis of epidemiological studies ^7^ revealed that breastfeeding decreased the risk of breast cancer. The systematic review conducted in Japan^13^ showed that breastfeeding possibly decreased that risk, but they did not report such an association in cohort studies.^13^

We aimed to elucidate the impact of reproductive factors on breast cancer incidence by conducting a pooled analysis of nine population‐based cohort studies in Japan.

## 2 METHODS

### 2.1 Study participants

The methods of the study were previously published in detail.^16^, ^17^, ^18^, ^19^ In this study, the study participants were females collected from nine population‐based cohort studies conducted in Japan: the Japan Public Health Center‐based Prospective Study, Cohort I (JPHC‐I),^11^ Cohort II (JPHC‐II),^11^ the Japan Collaborative Cohort Study (JACC),^10^ the Miyagi Cohort Study (MIYAGI‐I),^12^ the Three‐Prefecture Cohort Study in Miyagi (MIYAGI‐II),^15^, ^20^ the Three‐Prefecture Cohort Study in Aichi (AICHI),^15^, ^20^ the Takayama Study (TAKAYAMA),^21^ the Ohsaki National Health Insurance Cohort Study (OHSAKI),^22^ and the Life Span Study (LSS).^23^ The relevant ethics review committee approved each study. Details on informed consent were previously published.^10^, ^12^, ^20^, ^22^, ^23^, ^24^, ^25^ Since JPHC, JACC, MIYAGI‐I, MIYAGI‐II, and AICHI published the results of analyses on the same topic,^10^, ^11^, ^12^, ^15^ we used the latest version of the dataset and re‐analyzed the study results.

### 2.2 Exposures

We categorized six reproductive factors as follows: age at menarche [≤ 12, 13–14, 15–16, ≥17 years], age at first birth [≤20, 21–25, 26–30, 31–35, ≥36 years], number of births [nulliparous, 1, 2, ≥3], age at menopause [≤44, 45–49, 50–54, ≥55 years], use of female hormones [never, ever] (for cohort studies excluding MIYAGI‐II, AICHI, LSS), and breastfeeding history [never, ever] (for cohort studies excluding JACC, MIYAGI‐II, AICHI, Takayama).

### 2.3 Statistical analyses

In each population‐based cohort study, patients were excluded if (i) they had breast cancer history at baseline, (ii) their menopausal status at baseline was missing, or (iii) they were exposed to estimated radiation doses due to the atomic bomb of 100 mGy or more (for LSS only). Person years were calculated from the baseline date till the date of breast cancer diagnosis, date of death, and lost to follow‐up or the end of the study follow‐up, whichever occurred earliest.

We conducted analyses according to the menopausal status at baseline and according to the menopausal status at breast cancer diagnosis. Because none of the studies collected information on menopausal status after baseline, we hypothesized that women who were in premenopausal status at baseline became postmenopausal when they passed their 51st birthday. We set a cut point at age 51 based on prior studies.^16^, ^26^, ^27^ For women younger than 51 years old at the date of censoring who were reported to not be postmenopausal at baseline, we considered years of observation as the premenopausal period. For women who were 51 years old or older and/or were reported to be postmenopausal at baseline, we considered years of observation as the postmenopausal period. If women who were reported to not be postmenopausal at baseline became 51 years old during their observation period, we divided their years of observation into premenopausal period and postmenopausal period according to the 51st birthday.^16^, ^26^, ^27^

For all population‐based cohort studies, we calculated hazard ratio (HR) and 95% confidence interval (CI) by applying the Cox proportional‐hazards model. The reference category of each reproductive factor was defined as follows: ≤12 years (age at menarche), 21–25 years (age at first birth), nulliparous (number of births), ≤44 years (age at menopause), never (use of female hormones), and never (breastfeeding history). We applied two different models: model 1 in which we adjusted age and area (for multicentric studies including JPHC‐I, JPHC‐II, JACC, and LSS), model 2 in which we adjusted age, area (for multicentric studies including JPHC‐I, JPHC‐II, JACC, and LSS), history of smoking [never, former, current], body mass index (BMI) [<18.5, 18.5‐<23, 23‐<25, ≥25], history of drinking [nondrinker, occasional drinker (one to three times a month or less than once a week), one to four times a week, current drinker (more than five times a week)], environmental tobacco smoke (ETS) exposure during childhood [yes, no] (for studies excluding TAKAYAMA and LSS), environmental tobacco smoke (ETS) exposure at home and/or at work [yes, no] (for studies excluding TAKAYAMA and LSS), and mutually adjusted by each reproductive factor. Model 2 was the primary analytic model for the present study. Analyses on age at first birth were conducted among parous women. Age at menopause was not adjusted in the analyses on premenopausal at baseline or premenopausal at diagnosis. We created indicator terms for missing data of categorical variables. We also estimated the increase of breast cancer incidence per category of each reproductive factor by calculating P value for trend.

In each population‐based cohort study, the cohort‐specific hazard ratio and 95% CI were calculated. Then, they were combined by applying the random‐effects model.^28^ Heterogeneity between studies was evaluated by calculating I^2^‐statistic.^29^

We conducted analyses by using SAS statistical software package version 9.3 (JPHC‐I, JPHC‐II) or version 9.4 (MIYAGI‐I, MIYAGI‐II, TAKAYAMA, and OHSAKI), SPSS Statistics version 25.0 (JACC), Stata/MP 14.2 (AICHI), Stata/SE 15.1 (LSS), and Stata/MP 16.0 (random‐effects model). *p* values were two‐sided, and we considered *p* value <0.05 as statistically significant.

## 3 RESULTS

The baseline information of nine studies included in our analyses was summarized (Table 1). 187,999 women from nine population‐based cohorts were included: 61,113 women (32.5%) who were premenopausal at baseline and 126,886 women (67.5%) who were postmenopausal at baseline. The total breast cancer cases were 873 and 1456 for premenopausal and postmenopausal cancer, respectively.

**TABLE 1:** Characteristics of the cohort studies in the present pooled analysis

Study	Population	Age range at baseline, y	Follow‐up (start‐end)	For the present pooled analysis (Women only)						
				Age mean, y	Latestfollow‐up time	Mean follow‐up period, y	Menopausal statusat baseline (mean age, y)		Number of cases	
							Pre‐menopausal	Post‐menopausal	Pre‐menopausal	Post‐menopausal
JPHC‐I^†^	Japanese residents of five public health center areas in Japan	40–59	1990–2010	49.60	2013/12/31	21.30	9822 (44.72)	11629 (53.72)	203	207
JPHC‐II	Japanese residents of six public health center areas in Japan	40–69	1993–2010	53.42	2013/12/31	17.92	11986 (44.51)	19875 (58.79)	159	195
JACC^§^	Residents from 45 areas throughout Japan	40–79	1988–2001	58.20	2009/12/31	13.02	7821 (44.30)	30082 (61.90)	79	223
MIYAGI‐I	Residents of 14 municipalities in Miyagi Prefecture, Japan	40–64	1990–2007	51.69	2014/12/31	22.03	9127 (45.10)	11985 (56.72)	246	253
MIYAGI‐II	Residents of three municipalities in Miyagi Prefecture, Japan	40‐	1984–1992	56.76	1992/12/31	7.72	5065 (47.82)	9583 (61.53)	35	69
AICHI	Residents of two municipalities in Aichi Prefecture, Japan	40–103	1985–2000	56.57	2000/12/31	11.81	5846 (45.78)	11081 (62.27)	62	106
TAKAYAMA	Residents of Takayama city, Gifu Prefecture, Japan	35–101	1992–2008	56.07	2008/3/31	13.91	6373 (43.04)	9801 (63.69)	38	143
OHSAKI	Residents of 14 municipalities in Miyagi Prefecture, Japan	40–79	1994–2005	59.79	2008/3/31	10.94	4217 (45.97)	16088 (63.41)	50	161
LSS^¶^	Atomic bomb survivors in Hiroshima and Nagasaki, Japan	46–104	1991–2003	65.00	2003/12/31	10.80	856 (48.57)	6762 (67.08)	1	99
Total							61113	126886	873	1456

Abbreviations: AICHI, The Three‐Prefecture Cohort Study in Aichi; JACC, The Japan Collaborative Cohort Study; JPHC, The Japan Public Health Center‐based prospective Study; LSS, Life Span Study; MIYAGI‐I, The Miyagi Cohort Study; MIYAGI‐II, The Three‐Prefecture Cohort Study in Miyagi; OHSAKI, The Ohsaki National Health Insurance Cohort Study; TAKAYAMA, The Takayama Study

^†^ In JPHC‐I, subjects of one public health center area were excluded due to lack of incidence data.

^§^ In JACC, selected 22 areas with cancer incidence follow‐up data were used in this analysis.

^¶^ LSS originally started in 1950. This analysis included subjects who responded to both the 1978 and the 1991 surveys.

Table 2 describes the results of analyses according to menopausal status at the baseline. Among premenopausal women at baseline, model 1 showed that age at menarche, age at first birth, number of births, and use of female hormones were not associated with breast cancer incidence (P for trend: 1.00, 0.12, 0.05, and 0.10, for age at menarche, age at first birth, number of births, and use of female hormones, respectively). Model 1 also showed that breastfeeding history significantly decreased breast cancer incidence (HR: 0.78, 95% CI: 0.61–1.00). Model 2 showed that age at menarche, use of female hormones, and breastfeeding history were not associated with breast cancer incidence (P for trend: 0.76, 0.14, and 0.65, for age at menarche, use of female hormones, and breastfeeding history, respectively). Although P value for trend was not significant for age at first birth and number of births (P for trend: 0.15 and 0.30, respectively), women giving birth for the first time at ages ≥36 experienced significantly higher breast cancer incidence than at ages 21–25 years (Adjusted HR: 2.30, 95% CI: 1.39–3.79), and women who had 2 or ≥3 births experienced significantly lower breast cancer incidence than nulliparous women (Adjusted HR: 0.39, 95% CI: 0.19–0.81 and 0.28, 95% CI: 0.15–0.53, for 2 and ≥3 births, respectively).

**TABLE 2:** Reproductive factors and breast cancer risk according to menopausal status at baseline.

	Number of subjects (n=)	Person Years	Number of Cases (n=)	Model 1^†^					Model 2^§^				
				HR*	95% CI*	Heterogeneity		*p* for trend	HR*	95% CI*	Heterogeneity		*p* for trend
						I^2^ (%)	*p*				I^2^ (%)	*p*	
Premenopausal women													
Age at menarche													
≤12	8943	132652.6	129	Reference					Reference				
13–14	29733	437791.1	489	1.06	0.87–1.30	0.0	0.45		1.10	0.90–1.35	0.0	0.53	
15–16	14081	208713.7	202	0.95	0.75–1.20	0.0	0.61		1.00	0.79–1.27	0.0	0.59	
≥17	2641	40047.3	34	0.93	0.54–1.60	41.0	0.12	1.00	0.96	0.57–1.60	32.3	0.18	0.76
Age at first birth^¶^													
≤20	2958	47971.8	35	0.81	0.58–1.15	0.0	0.81		0.81	0.57–1.15	0.0	0.90	
21–25	32991	494463.9	485	Reference					Reference				
26–30	15972	226166.9	238	1.10	0.92–1.32	14.9	0.31		1.07	0.88–1.30	21.2	0.25	
31–35	2314	32745.2	41	1.39	1.01–1.92	0.0	0.66		1.24	0.89–1.73	0.0	0.67	
≥36	645	9549.1	19	2.69	1.57–4.59	19.4	0.29	0.12	2.30	1.39–3.79	0.0	0.46	0.15
Number of births													
Nulliparous	2821	38720.0	67	Reference					Reference				
One	3640	53675.8	72	0.73	0.52–1.03	0.0	0.79		0.50	0.22–1.13	26.1	0.23	
Two	20663	319145.7	328	0.53	0.41–0.70	0.0	0.74		0.39	0.19–0.81	21.4	0.27	
More than three	22205	319953.3	314	0.45	0.34–0.59	0.0	0.83	0.05	0.28	0.15–0.53	0.0	0.65	0.30
Use of female hormones													
Never	41982	653631.3	690	Reference					Reference				
Ever	4531	78728.2	93	1.22	0.96–1.55	8.3	0.36	0.10	1.20	0.94–1.52	5.6	0.38	0.14
Breastfeeding history													
Never	3735	70074.8	75	Reference					Reference				
Ever	29375	562441.3	534	0.78	0.61–1.00	0.0	0.47	0.05	0.94	0.71–1.24	0.0	0.75	0.65
Postmenopausal women													
Age at menarche													
≤12	5681	68191.8	70	Reference					Reference				
13–14	36535	426582.0	453	0.97	0.76–1.26	0.0	0.79		0.99	0.77–1.28	0.0	0.79	
15–16	47051	536820.1	505	0.85	0.66–1.10	0.0	0.51		0.88	0.68–1.14	2.0	0.42	
≥17	25672	296025.7	241	0.78	0.59–1.03	0.0	0.90	0.47	0.82	0.62–1.08	0.0	0.92	0.17
Age at first birth^¶^													
≤20	8748	105549.8	65	0.86	0.67–1.12	0.0	0.76		0.88	0.68–1.14	0.0	0.76	
21–25	63813	761635.6	604	Reference					Reference				
26–30	29440	332021.8	400	1.47	1.29–1.67	0.0	0.56		1.38	1.21–1.58	0.0	0.76	
31–35	4355	48847.8	65	1.80	1.37–2.37	6.6	0.38		1.52	1.16–2.00	0.0	0.45	
≥36	1167	13557.9	17	1.92	1.18–3.12	0.0	0.66	<0.01	1.48	0.89–2.44	0.0	0.55	0.30
Number of births													
Nulliparous	6802	71902.9	95	Reference					Reference				
One	7548	90537.4	134	0.94	0.72–1.25	0.0	0.79		1.14	0.68–1.89	0.0	0.90	
Two	28574	377216.9	378	0.63	0.48–0.84	23.3	0.25		0.81	0.49–1.33	0.0	0.77	
More than three	60005	626936.5	517	0.47	0.33–0.65	47.4	0.08	0.17	0.63	0.39–1.04	0.0	0.56	0.03
Age at menopause													
≤44	15645	192259.3	161	Reference					Reference				
45–49	36784	441502.6	394	1.09	0.88–1.34	17.5	0.29		1.12	0.90–1.40	19.0	0.27	
50–54	52112	607618.3	614	1.23	1.00–1.52	21.3	0.25		1.27	1.02–1.57	22.6	0.24	
≥55	5735	61345.6	69	1.47	0.97–2.22	39.9	0.10	0.45	1.48	1.01–2.17	30.2	0.18	0.37
Use of female hormones													
Never	81141	966615.8	934	Reference					Reference				
Ever	7387	109707.2	95	0.97	0.79–1.21	0.0	0.79	0.80	0.94	0.76–1.17	0.0	0.80	0.60
Breastfeeding history													
Never	5212	83449.5	91	Reference					Reference				
Ever	55087	928418.0	700	0.66	0.51–0.86	21.8	0.28	<0.01	0.88	0.66–1.17	0.0	0.59	0.39

* CI, confidence interval; HR, hazard ratio.

^†^ Model 1: adjusted by age and area (for multicentric studies including JPHC‐I, JPHC‐II, JACC, and LSS).

^§^ Model 2: adjusted by age, area (for multicentric studies including JPHC‐I, JPHC‐II, JACC, and LSS), history of smoking [never, former, and current], Body Mass Index [<18.5, 18.5‐<23, 23‐<25, ≥25], history of drinking [nondrinker, occasional drinker (one to three times a month or less than once a week), one to four times a week, current drinker (more than five times a week)], environmental tobacco smoke (ETS) exposure during childhood [yes, no] (for studies excluding TAKAYAMA and LSS), environmental tobacco smoke (ETS) exposure at home and/or at work [yes, no] (for studies excluding TAKAYAMA and LSS), and mutually adjusted by age at menarche [≤12, 13–14, 15–16, ≥17], age at first birth [≤20, 21–25, 26–30, 31–35, ≥36], number of births [nulliparous, 1, 2, ≥3], use of female hormones [never, ever] (for studies including JPHC‐I, JPHC‐II, JACC, MIYAGI‐I, TAKAYAMA, and OHSAKI), and breastfeeding history [never, ever] (for studies including JPHC‐I, JPHC‐II, MIYAGI‐I, OHSAKI, and LSS).

^¶^ Analyses on age at first birth were conducted among parous women.

Among postmenopausal women at baseline, model 1 showed that higher age at first birth significantly increased breast cancer incidence (*p* for trend: <0.01), and breastfeeding history significantly decreased breast cancer incidence (HR: 0.66, 95% CI: 0.51–0.86). Model 1 also showed that age at menarche, number of births, age at menopause, and use of female hormones were not associated with breast cancer incidence (P for trend: 0.47, 0.17, 0.45, and 0.80, for age at menarche, number of births, age at menopause, and use of female hormones, respectively). Model 2 showed that more births significantly decreased breast cancer incidence (*p* for trend: 0.03). Model 2 also showed that age at menarche, use of female hormones, and breastfeeding history were not associated with breast cancer incidence (P for trend: 0.17, 0.60, and 0.39, for age at menarche, use of female hormones, and breastfeeding history, respectively). Although P value for trend was not significant on age at first birth and age at menopause (*p* for trend: 0.30 and 0.37, respectively), women giving first birth at ages 26–30 or 31–35 years experienced significantly higher breast cancer incidence than women giving first birth at ages 21–25 years (Adjusted HR: 1.38, 95% CI: 1.21–1.58 and 1.52, 95% CI: 1.16–2.00, for ages 26–30 and 31–35 years, respectively), and women whose age at menopause: 50–54 or ≥55 years experienced significantly higher breast cancer incidence than age at menopause: ≤44 years (Adjusted HR: 1.27, 95% CI: 1.02–1.57 and 1.48, 95% CI: 1.01–2.17, for 50–54 and ≥55 years, respectively).

Supplementary Table S1 describes the results of our analyses according to the menopausal status at the diagnosis of breast cancer. Results were mostly consistent with those presented in Table 2, excluding use of female hormones among premenopausal women and age at first birth among postmenopausal women. Female hormones significantly increased breast cancer incidence among premenopausal women at cancer diagnosis (Adjusted HR: 1.53, 95% CI: 1.04–2.25) in model 2. Higher age at first birth significantly increased the incidence of breast cancer among postmenopausal women at cancer diagnosis (*p* < 0.001) in model 2.

## 4 DISCUSSION

The present study targeted more than 180,000 Japanese women, and elucidated the association of reproductive factors with the incidence of breast cancer. Among premenopausal women, use of female hormones significantly increased premenopausal breast cancer. Although P value for trend was not significant for age at first birth and number of births, women giving first birth at ages ≥36 experienced significantly higher incidence and women who had ≥2 births experienced significantly lower incidence. Among postmenopausal women, more births significantly decreased breast cancer incidence. Although P value for trend was not significant for age at first birth and age at menopause, women giving first birth at ages 26–35 years and women with age at menopause ≥50 years experienced significantly higher breast cancer incidence. Breast cancer incidence was similar regardless of age at menarche or breastfeeding history among both premenopausal and postmenopausal women.

Lower age at menarche has been regarded as one of the risk factors of breast cancer.^6^, ^9^, ^11^, ^15^ Kelsey et al^6^ indicated that younger age of menarche increased breast cancer because of earlier onset of ovulatory cycles, longer period of exposure to estrogen or higher estrogen level for some years after menarche. However, we did not observe such an association in our study, which might be due to the small breast cancer cases (=34) in our study of women whose age at menarche: ≥17 years. Improved nutrition has resulted in lower age at menarche in Asian countries as well as in Western countries.^30^, ^31^, ^32^ In our study, the percentage of women whose age at menarche: ≥17 years old was 4.8% (premenopausal women at baseline) and 22.3% (postmenopausal women at baseline), which may have resulted in the failure to find a significant result. It is also speculated that breast cancer incidence is higher among women whose age at menarche: ≥17 years due to factors except for reproductive factors.

Significantly higher breast cancer incidence was observed in women with age at menopause: 50–54 and ≥55 years than those with age at menopause: ≤44 years. Although a previous meta‐analysis in Japan ^9^ compared women whose age at menopause: ≥50 years with women whose age at menopause ≤49 years, the present pooled analysis created another category of age at menopause (≥55 years). Previous studies have showed that longer exposure to female hormones may have resulted in higher breast cancer risk among women with higher age of menopause.^6^, ^33^

The association of parity with breast cancer incidence was also reported in previous studies.^11^, ^34^, ^35^, ^36^ Previous reports have showed that parity decreased breast cancer risk because differentiation of mammary gland epithelium was promoted by pregnancy, and these differentiated cells would be protected from neoplastic transformation.^6^, ^37^ The impact of parity on breast cancer was greater among postmenopausal women than among premenopausal women in our study, which may mean that parity has long‐term effects rather than short‐term effects.

In the present study, use of female hormones significantly elevated the incidence of breast cancer among premenopausal women. However, we did not observe this association among postmenopausal women. Few studies in Asia analyzed the impact of female hormones on breast cancer, mainly due to lower prevalence of such hormone use compared with Western countries.^11^, ^12^ In contrast, the results of our study are consistent with those from studies in Western countries.^38^, ^39^, ^40^, ^41^ A previous pooled analysis ^38^ reported significantly higher breast cancer risk among women who currently used female hormones or used them in the past 10 years, while such an association was not reported among women who had used female hormones ≥10 years ago. Another study ^41^ reported that use of female hormones significantly increased breast cancer risk among premenopausal women, but such a harmful effect declined among postmenopausal women. These findings may indicate that timing of the use of female hormones is important; use of female hormones mainly as oral contraceptives may increase the risk of breast cancer during the premenopausal period, but such an effect will not continue into the postmenopausal period.

We did not find an association between history of breastfeeding and breast cancer, and this result was inconsistent with some of the previous studies.^6^, ^7^, ^42^, ^43^ Possible mechanisms for such an association have been proposed, including hormonal changes (reduced estrogen and progesterone levels and increased prolactin level),^6^, ^44^ delaying re‐establishment of ovulation,^6^, ^44^ and excreting estrogens and carcinogens out of breast ducts.^44^ A pooled analysis of epidemiological studies ^7^ revealed that breastfeeding significantly decreased breast cancer incidence. In contrast, other epidemiological studies including our study did not find such an association.^11^, ^12^, ^13^, ^14^ The lack of statistical significance in our study could be explained by sample size; among nine cohort studies which were included in our pooled analysis, the information on breastfeeding was not available for four studies. Therefore, we could not include these four cohort studies in the analysis of breastfeeding, which could have resulted in the small breast cancer cases. Furthermore, we observed a significant reduction in breast cancer risk among breastfed postmenopausal women in the analysis adjusted by age and area (model 1). Because the cohort of premenopausal women may have included women who lived in the relatively recent era compared with the cohort of postmenopausal women, we speculate that duration of breastfeeding has become shorter in the recent era in Japan.

This study has four limitations: (1) we could not conduct pooled analyses according to hormone receptor or histology because this information was missing in some of the included cohort studies; (2) we could not evaluate the association between breast cancer risk and duration of female hormones’ use, type of female hormones including hormone replacement therapy (HRT), oral contraceptive (OC), or breastfeeding because most of included studies did not collect this information; (3) we could not calculate hazard ratio per 1‐year increase in age at menarche/menopause/first birth because several studies which were included in this pooled analysis collected this information as categorical variables, not as continuous variables; (4) there might be other unmeasured confounding factors that affected our study results.

In conclusion, in Japan, use of female hormones significantly increased premenopausal breast cancer, and greater number of births significantly decreased breast cancer risk in postmenopausal women. However, breast cancer risk was similar according to age at menarche/breastfeeding history in both premenopausal and postmenopausal women. Although some of reproductive factors studies in the present study are not modifiable factors, understanding high‐risk population would be important for preventing breast cancer including chemoprevention and early detection. Further studies would be needed to elucidate the impact of reproductive factors according to hormone receptors or histology.

## CONFLICT OF INTEREST

None declared.

## AUTHOR CONTRIBUTIONS

All the authors 1) have made substantial contributions to conception and design, or acquisition of data, or analysis and interpretation of data; 2) were involved in drafting the manuscript or revising it critically for important intellectual content; 3) gave final approval of the version to be published. Each author participated sufficiently in the work to take public responsibility for appropriate portions of the content; and 4) agreed to be accountable for all aspects of the work in ensuring that questions related to the accuracy or integrity of any part of the work are appropriately investigated and resolved.

## Supporting information

Supplementary Material

## Data Availability

The datasets used in the manuscript are not publicly available. A collaboration with each cohort study groups would be required to access the datasets.
