# Supplementary material for: Impact of reproductive factors on breast cancer incidence: Pooled analysis of nine cohort studies in Japan
Source: Cancer Med. 2021 Mar 1;10(6):2153–63. doi: 10.1002/cam4.3752 (PMC7957169; doi:10.1002/cam4.3752)
Supplement: Supplementary file 1 — Supplementary Material [file CAM4-10-2153-s001.docx]

**Supplementary table 1. Reproductive factors and breast cancer risk according to menopausal status at breast cancer diagnosis**

|  | Number of  subjects (n=) | Person  Years | Number of  Cases (n=) | Model 1 | | | | | | | Model 2 | | | | | | |
| --- | --- | --- | --- | --- | --- | --- | --- | --- | --- | --- | --- | --- | --- | --- | --- | --- | --- |
|  |  |  |  | HR | 95%CI | | | Heterogeneity | | P for trend | HR | 95%CI | | | Heterogeneity | | P for trend |
|  |  |  |  |  |  |  |  |  |  |  |  |  |  |  |  |  |  |
|  |  |  |  |  |  |  |  | I^2^ (%) | P |  |  |  |  |  | I^2^ (%) | P |  |
| **Premenopausal women** |  |  |  |  |  |  |  |  |  |  |  |  |  |  |  |  |  |
| Age at menarche |  |  |  |  |  |  |  |  |  |  |  |  |  |  |  |  |  |
| ≤ 12 | 8061 | 47752.2 | 54 | Reference |  |  |  |  |  |  | Reference |  |  |  |  |  |  |
| 13-14 | 26155 | 128807.7 | 140 | 0.87 | 0.60 | - | 1.25 | 15.0 | 0.32 |  | 0.92 | 0.63 | - | 1.33 | 14.4 | 0.32 |  |
| 15-16 | 12030 | 48885.6 | 42 | 0.76 | 0.43 | - | 1.35 | 33.2 | 0.19 |  | 0.81 | 0.47 | - | 1.39 | 25.4 | 0.24 |  |
| ≥ 17 | 2012 | 6892.4 | 7 | 2.33 | 0.90 | - | 6.03 | 0.0 | 0.97 | 0.97 | 2.67 | 1.00 | - | 7.10 | 0.0 | 0.99 | 0.83 |
| Age at first birth¶ |  |  |  |  |  |  |  |  |  |  |  |  |  |  |  |  |  |
| ≤ 20 | 2480 | 12666.5 | 9 | 0.91 | 0.46 | - | 1.83 | 0.0 | 0.51 |  | 0.81 | 0.40 | - | 1.65 | 0.0 | 0.61 |  |
| 21-25 | 28275 | 137907.3 | 157 | Reference |  |  |  |  |  |  | Reference |  |  |  |  |  |  |
| 26-30 | 14201 | 67148.8 | 67 | 0.86 | 0.56 | - | 1.32 | 41.8 | 0.10 |  | 0.80 | 0.51 | - | 1.23 | 42.5 | 0.10 |  |
| 31-35 | 2061 | 10362.9 | 12 | 1.30 | 0.72 | - | 2.37 | 0.0 | 0.76 |  | 1.15 | 0.62 | - | 2.13 | 0.0 | 0.75 |  |
| ≥ 36 | 569 | 2613.8 | 6 | 4.70 | 2.03 | - | 10.88 | 0.0 | 0.98 | 0.71 | 4.12 | 1.54 | - | 10.97 | 0.0 | 0.44 | 0.61 |
| Number of births |  |  |  |  |  |  |  |  |  |  |  |  |  |  |  |  |  |
| nulliparous | 2435 | 11700.5 | 12 | Reference |  |  |  |  |  |  | Reference |  |  |  |  |  |  |
| one | 3095 | 14756.2 | 27 | 1.68 | 0.82 | - | 3.46 | 0.0 | 0.82 |  | 2.18 | 0.55 | - | 8.64 | 0.0 | 0.70 |  |
| two | 17447 | 82798.0 | 91 | 0.77 | 0.40 | - | 1.46 | 0.0 | 0.77 |  | 0.83 | 0.21 | - | 3.33 | 0.0 | 0.91 |  |
| more than three | 19276 | 78122.7 | 91 | 0.71 | 0.35 | - | 1.46 | 0.0 | 0.75 | 0.35 | 0.68 | 0.13 | - | 3.58 | 0.0 | 0.68 | 0.26 |
| Use of female hormones |  |  |  |  |  |  |  |  |  |  |  |  |  |  |  |  |  |
| never | 37080 | 179005.2 | 198 | Reference |  |  |  |  |  |  | Reference |  |  |  |  |  |  |
| ever | 4158 | 21226.6 | 33 | 1.56 | 1.07 | - | 2.29 | 0.0 | 0.95 | 0.02 | 1.53 | 1.04 | - | 2.25 | 0.0 | 0.96 | 0.03 |
| Breastfeeding history |  |  |  |  |  |  |  |  |  |  |  |  |  |  |  |  |  |
| never | 3346 | 17669.9 | 13 | Reference |  |  |  |  |  |  | Reference |  |  |  |  |  |  |
| ever | 23933 | 130711.1 | 118 | 1.12 | 0.62 | - | 2.02 | 0.0 | 0.99 | 0.71 | 1.17 | 0.64 | - | 2.13 | 0.0 | 0.96 | 0.60 |
| **Postmenopausal women** |  |  |  |  |  |  |  |  |  |  |  |  |  |  |  |  |  |
| Age at menarche |  |  |  |  |  |  |  |  |  |  |  |  |  |  |  |  |  |
| ≤ 12 | 13985 | 153017.0 | 143 | Reference |  |  |  |  |  |  | Reference |  |  |  |  |  |  |
| 13-14 | 64278 | 736671.5 | 809 | 1.13 | 0.94 | - | 1.35 | 0.0 | 0.78 |  | 1.15 | 0.94 | - | 1.40 | 0.0 | 0.78 |  |
| 15-16 | 60370 | 701029.6 | 672 | 0.98 | 0.81 | - | 1.18 | 0.0 | 0.47 |  | 1.02 | 0.83 | - | 1.26 | 0.0 | 0.44 |  |
| ≥ 17 | 28218 | 331562.8 | 269 | 0.84 | 0.68 | - | 1.05 | 0.0 | 0.75 | 0.04 | 0.91 | 0.71 | - | 1.15 | 0.0 | 0.86 | 0.15 |
| Age at first birth¶ |  |  |  |  |  |  |  |  |  |  |  |  |  |  |  |  |  |
| ≤ 20 | 11545 | 142143.9 | 91 | 0.84 | 0.67 | - | 1.04 | 0.0 | 0.60 |  | 0.90 | 0.71 | - | 1.14 | 0.0 | 0.71 |  |
| 21-25 | 94596 | 1122827.0 | 939 | Reference |  |  |  |  |  |  | Reference |  |  |  |  |  |  |
| 26-30 | 44293 | 492321.7 | 573 | 1.37 | 1.22 | - | 1.54 | 16.7 | 0.29 |  | 1.34 | 1.19 | - | 1.51 | 1.0 | 0.43 |  |
| 31-35 | 6492 | 71633.6 | 95 | 1.63 | 1.30 | - | 2.03 | 4.2 | 0.40 |  | 1.47 | 1.16 | - | 1.86 | 0.0 | 0.47 |  |
| ≥ 36 | 1777 | 20601.2 | 30 | 2.00 | 1.39 | - | 2.89 | 0.0 | 0.61 | <0.001 | 1.68 | 1.13 | - | 2.49 | 0.0 | 0.66 | <0.001 |
| Number of births |  |  |  |  |  |  |  |  |  |  |  |  |  |  |  |  |  |
| nulliparous | 9439 | 100712.9 | 150 | Reference |  |  |  |  |  |  | Reference |  |  |  |  |  |  |
| one | 10959 | 130144.7 | 180 | 0.82 | 0.63 | - | 1.06 | 17.3 | 0.29 |  | 0.85 | 0.54 | - | 1.35 | 0.0 | 0.91 |  |
| two | 47936 | 614523.7 | 619 | 0.60 | 0.46 | - | 0.80 | 47.9 | 0.06 |  | 0.65 | 0.42 | - | 1.00 | 0.0 | 0.97 |  |
| more than three | 81001 | 875162.2 | 748 | 0.47 | 0.34 | - | 0.64 | 60.7 | 0.01 | 0.10 | 0.52 | 0.34 | - | 0.81 | 0.0 | 0.78 | 0.02 |
| Age at menopause |  |  |  |  |  |  |  |  |  |  |  |  |  |  |  |  |  |
| ≤ 44 | 15645 | 192259.2 | 161 | Reference |  |  |  |  |  |  | Reference |  |  |  |  |  |  |
| 45-49 | 36784 | 441502.5 | 394 | 1.08 | 0.87 | - | 1.34 | 21.3 | 0.25 |  | 1.12 | 0.90 | - | 1.39 | 21.3 | 0.25 |  |
| 50-54 | 73921 | 913991.4 | 875 | 1.23 | 0.98 | - | 1.54 | 34.0 | 0.15 |  | 1.26 | 1.01 | - | 1.59 | 32.9 | 0.15 |  |
| ≥ 55 | 5735 | 61345.6 | 69 | 1.46 | 0.97 | - | 2.19 | 39.9 | 0.10 | 0.53 | 1.48 | 1.01 | - | 2.16 | 30.8 | 0.17 | 0.47 |
| Use of female hormones |  |  |  |  |  |  |  |  |  |  |  |  |  |  |  |  |  |
| never | 121484 | 1443546.0 | 1438 | Reference |  |  |  |  |  |  | Reference |  |  |  |  |  |  |
| ever | 11790 | 167422.3 | 156 | 1.01 | 0.85 | - | 1.20 | 0.0 | 0.67 | 0.90 | 1.01 | 0.84 | - | 1.21 | 0.0 | 0.67 | 0.91 |
| Breastfeeding history |  |  |  |  |  |  |  |  |  |  |  |  |  |  |  |  |  |
| never | 8912 | 135359.6 | 152 | Reference |  |  |  |  |  |  | Reference |  |  |  |  |  |  |
| ever | 83706 | 1361292.0 | 1116 | 0.64 | 0.48 | - | 0.86 | 62.5 | 0.03 | <0.01 | 0.88 | 0.71 | - | 1.09 | 0.0 | 0.74 | 0.24 |

*HR, hazard ratio; CI, confidence interval.

†Model 1: adjusted by age and area (for multi-centric studies including JPHC-I, JPHC-II, JACC, LSS).

§Model 2: adjusted by age, area (for multi-centric studies including JPHC-I, JPHC-II, JACC, LSS), history of smoking [never, former, current], Body Mass Index [<18.5, 18.5-<23, 23-<25, ≥25], history of drinking [nondrinker, occasional drinker (1-3 times a month or less than once a week), 1-4 times a week, current drinker (more than 5 times a week)], environmental tobacco smoke (ETS) exposure during childhood [yes, no] (for studies excluding TAKAYAMA and LSS), environmental tobacco smoke (ETS) exposure at home and/or at work [yes, no] (for studies excluding TAKAYAMA and LSS), and mutually adjusted by age at menarche [≤12, 13-14, 15-16, ≥17], age at first birth [≤20, 21-25, 26-30, 31-35, ≥36], number of births [nulliparous, 1, 2, ≥3], use of female hormones [never, ever] (for studies including JPHC-I, JPHC-II, JACC, MIYAGI-I, TAKAYAMA, OHSAKI), breastfeeding history [never, ever] (for studies including JPHC-I, JPHC-II, MIYAGI-I, OHSAKI, LSS).

¶Analyses on age at first birth were conducted among parous women.
